# Implementation of a community-based intervention in the most rural and remote districts of Zambia: a process evaluation of safe motherhood action groups

**DOI:** 10.1186/s13012-018-0766-1

**Published:** 2018-05-31

**Authors:** Choolwe Jacobs, Charles Michelo, Mosa Moshabela

**Affiliations:** 1 School of Nursing and Public Health, University of KwaZulu-Natal, Durban, South Africa; 2 School of Public Health, Department of Epidemiology and Biostatistics, University of Zambia, Lusaka, Zambia; 3 Strategic Centre for Health Systems Metrics and Evaluations (SCHEME), School of Public Health, University of Zambia, Lusaka, Zambia; 4 Africa Health Research Institute, KwaZulu Natal, South Africa

**Keywords:** Access to healthcare, Process evaluation, Maternal health, Neonatal health, Remote and rural areas, Lay health workers, Zambia

## Abstract

**Background:**

A community-based intervention known as Safe Motherhood Action Groups (SMAGs) was implemented to increase coverage of maternal and neonatal health (MNH) services among the poorest and most remote populations in Zambia. While the outcome evaluation demonstrated statistically significant improvement in the MNH indicators, targets for key indicators were not achieved, and reasons for this shortfall were not known. This study was aimed at understanding why the targeted key indicators for MNH services were not achieved.

**Methods:**

A process evaluation, in accordance with the Medical Research Council (MRC) framework, was conducted in two selected rural districts of Zambia using qualitative approaches. Focus group discussions were conducted with SMAGs, volunteer community health workers, and mothers and in-depth interviews with healthcare providers. Content analysis was done.

**Results:**

We found that SMAGs implemented much of the intervention as was intended, particularly in the area of women’s education and referral to health facilities for skilled MNH services. The SMAGs went beyond their prescribed roles to assist women with household chores and personal problems and used their own resources to enhance the success of the intervention. Deficiencies in the intervention were reported and included poor ongoing support, inadequate supplies and lack of effective transportation such as bicycles needed for the SMAGs to facilitate their work. Factors external to the intervention, such as inadequacy of health services and skilled healthcare providers in facilities where SMAGs referred mothers and poor geographical access, may have led SMAGs to engage in the unintended role of conducting deliveries, thus compromising the outcome of the intervention.

**Conclusion:**

We found evidence suggesting that although SMAGs continue to play pivotal roles in contribution towards accelerated coverage of MNH services among hard-to-reach populations, they are unable to meet some of the critical sets of MNH service-targeted indicators. The complexities of the implementation mechanisms coupled with the presence of setting specific socio-cultural and geographical contextual factors could partially explain this failure. This suggests a need for innovating existing implementation strategies so as to help SMAGs and any other community health system champions to effectively respond to MNH needs of most-at-risk women and promote universal health coverage targeting hard-to-reach groups.

**Electronic supplementary material:**

The online version of this article (10.1186/s13012-018-0766-1) contains supplementary material, which is available to authorized users.

## Background

Zambia is one of the countries in the Sub-Saharan region with poor maternal outcomes [1, 2]. Despite reductions in maternal and neonatal mortality rates in Zambia, approximately 398 deaths per 100,000 live births and 24 per 1000 live births were reported in the recent Zambia Demographic Health Survey (ZDHS) [1]. Arguably, most of these deaths could be averted if all women utilised the maternal and neonatal health (MNH) services [3]. Unfortunately, in many developing countries similar to Zambia, poor women living in the most remote and rural areas are least likely to receive adequate MNH care, including antenatal care (ANC), skilled birth attendance (SBA) and postnatal Care (PNC), due to service utilisation challenges such as geographical and socio-cultural barriers [4–8]. In the most remote and rural areas of Zambia, only a third of women receive SBA and PNC [4]. There is a need to improve the utilisation of and access to MNH care services for women, particularly those in rural areas. Increasingly, community-based interventions through community health workers (CHWs) have been receiving recognition as an effective strategy to improve utilisation and access related to health facility-based services [9–11]. By serving as a linkage between the community and the formal health system, CHWs are well-placed to bridge the service delivery gap in poor-resource settings [10], often through effective referral to health services [12, 13]. Zambia has also been responding to the challenges of poor access and utilisation of MNH services through community-based interventions, and among them are the Safe Motherhood Action Groups (SMAGs).

### Description of the community-based intervention

In 2013, through the Health for the Poorest Population (HPP) programme, a community-based intervention was designed to strengthen MNH services. Through this project, intensified efforts were made to reduce disparities in MNH services through SMAGs. The goal was to make pregnancy safer through accelerated delivery of priority MNH interventions (ANC, SBA and PNC). The SMAGs, although not scaled up in all the districts, have been in existence in Zambia since 2003. The aim of the SMAGs programme was to raise awareness about pregnancy and birth-related complications and to reduce critical delays in decision-making at a household level about seeking life-saving maternal healthcare in health facilities [14]. The programme was also aimed at improving access to MNH services through linkages between the community and the healthcare facility [14]. Specifically, in the HPP project, the aim of the SMAG intervention was to reduce disparities in intervention coverage for MNH services and help meet the national targets (80 and 60% for ANC at least four times and SBA, respectively) for coverage among the remote and poorest populations in rural areas.

The SMAGs are groups of women and men working as CHWs, traditional birth attendants (TBAs), child health promoters or growth monitoring promoters, malaria agents and lay counsellors. The SMAGs were recruited and trained in safe motherhood skills. The choice and recruitment of SMAGs were guided by the Zambia Ministry of Health standards that entail one CHW for every 500 of the population. A standard training programme of 5 days was used to empower SMAGs with safe motherhood knowledge and skills, specifically for promoting antenatal care, delivery in a health facility with a skilled provider, postnatal home visits and essential neonatal care. SMAGs were specifically trained in focused antenatal care to identify danger signs, encourage women to start ANC early, attend ANC at least four times and receive skilled deliveries. In addition, SMAGs were trained in essential newborn care, including the provision of effective cord care, early initiation of exclusive breastfeeding and reporting maternal and neonatal deaths that in the community.

The key roles SMAGs played in the intervention were to refer women for ANC, delivery and complications during pregnancy, delivery and the postnatal period; actively following up women to close the gap in the MNH continuum of care; and providing facility-based birth preparedness messages to pregnant women and their spouses in the community. The work by the SMAGs was voluntary, and they could be called upon anytime a woman in the community needed their services. The Neighbourhood Health Committees and health facility staff, including district coordinators, supervised the implementation of the SMAG activities.

### Outcomes of the community-based intervention

An outcome evaluation was conducted based on household survey data collected at three time points during the implementation of the intervention: baseline, mid-point (mid-line) and end-point (end-line).

Observations from the outcome evaluation showed a statistically significant effect of the deployment of the SMAG intervention on most of the MNH outcomes of interest, ANC, SBA and PNC, with an increasing trend over time. Although statistically significant, the increase in coverage over time was programmatically marginal, and the programme’s targets for coverage were not met (42 versus 80% for focused ANC, and 49 versus 60% for SBA) except for PNC for within 48 h by SMAGs (22 versus 20%). This study was therefore aimed at understanding why the targeted key indicators for MNH services were not achieved.

There were also variations at the district level in certain outcome indicators of interest, such as postnatal care, the details of which were reported by Jacobs et al. [15] on the outcome evaluation of the intervention. Similar to the findings in other community-based intervention studies [16–18], the observed results of the outcome evaluation needed further explanation, taking into account interactions between contextual factors and the SMAG intervention. Therefore, the purpose of this paper was to understand why the targeted key indicators for MNH services were not achieved. Specifically, the paper explored the following questions: (1) Was the intervention (the SMAGs) implemented as it was intended? (2) What are the factors external to the intervention that may have influenced implementation of the intervention? and (3) What are the possible mechanisms that likely explain the gap between achieved and targeted outcomes? This study will provide an understanding of possible explanations for the partial success of the SMAG intervention, taking into account the contextual factors under implementations occurred, so as to inform similar future programmatic decision-making.

## Methods

### Study setting

The study was conducted in two remote districts, located in Luapula and the northern provinces of Zambia. The districts are among the four districts for the HPP where the SMAGs programme was implemented [15]. To select the study districts, we first stratified the districts into two provinces. Within each of the two provinces, we randomly selected one of the two intervention districts by flipping a coin. From each of the selected districts, two intervention health facilities were randomly selected using a lottery method, where all the facilities were assigned numbers, after which two numbers were selected at random.

### Design

A process evaluation was conducted retrospectively, in accordance with the Medical Research Council (MRC) framework [19, 20], between November 2016 and January 2017, using focus group discussions (FDGs) and in-depth interviews (IDIs). Process evaluations have been reported as an essential part of community-based interventions [19, 21], needed to provide insight on how well programme activities are implemented, and performing within the context in which implementation occurs [22]. According to Moore and colleagues [19], effect sizes alone may not inform policy and implementers on how such community-based interventions may be replicated or reproduced in specific contexts. Moore et al. further argued that process evaluations are needed to assess fidelity and quality of implementation, as well as to identify causal mechanisms and contextual factors associated with the variations in the outcomes of interventions.

### Key constructs of the process evaluation

**Fig. 1:**
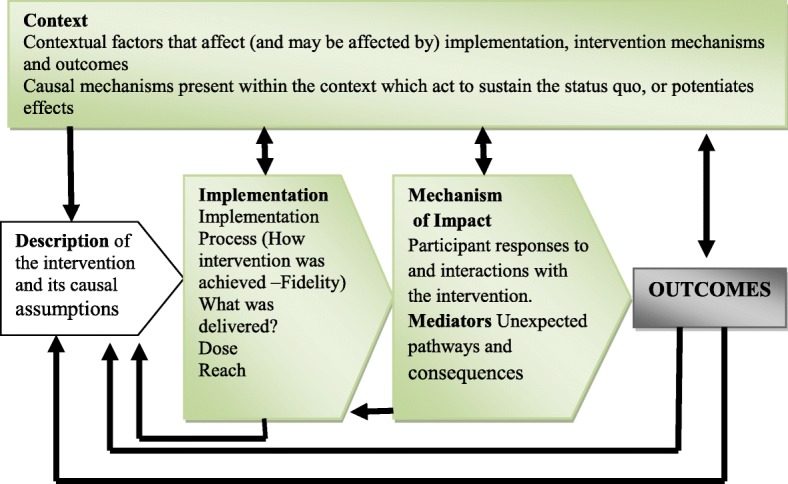
Key constructs of the process evaluation and the relations among the constructs [19]

The UK MRC framework [21] was adopted to guide the identification of relevant key constructs and to generate evaluation questions in this study. According to Moore et al. [19], despite a need to understand casual assumptions that underpin an intervention in complex interventions such as the HPP project, there is also a need to understand how the intervention worked by scrutinising its plausibility and the relations between implementation, mechanisms of impact and context. The SMAG intervention was regarded as complex because it comprised multiple interacting components and a number of targets to be met. According to the MRC framework, an intervention may have limited effects or positive outcomes due to its implementation processes such as fidelity, whether the intervention was implemented as intended or the degree to which an intervention is delivered as intended; the dose; the quantity of the intervention implemented; and its reach, whether the intended audience comes into contact with the intervention or not [23]. While the implementation context includes anything external to the intervention that may act as a barrier or facilitator to its implementation [19]. Further, the mechanism of impact guides an understanding of how an intervention was delivered and how the effects of the intervention occurred. An illustration of these key constructs and the assumptions on their interaction with the intervention is provided in Fig. 1.

Existing evidence shows that the outcome of a complex community-based intervention can be influenced by the interactions between the stated three key constructs, namely implementation, context and mechanisms [19, 21].

### Causal assumptions for the intervention

**Fig. 2:**
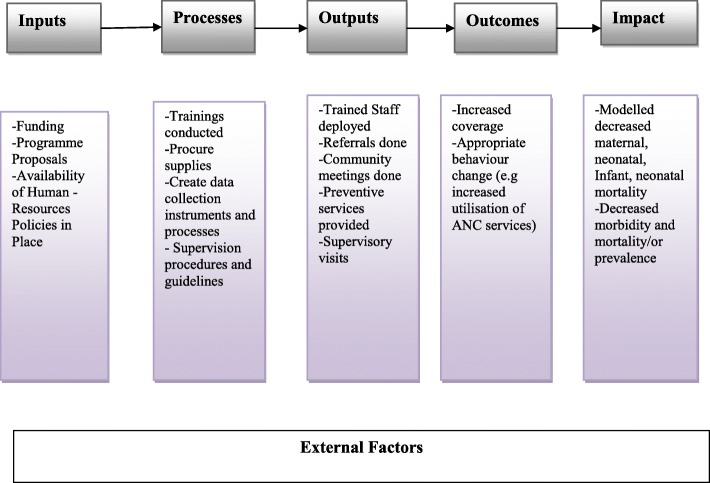
Logical model for the Health for the Poorest Populations project

The logic ‘inputs-processes-outputs-outcomes-impact model’ was used as a theory of change to guide the implementation of the intervention. Figure 2 describes the inputs, outputs (activities, participation) and their links to outcomes. Based on the model, the inputs included implementation plans, human resources, funding and working with district health teams. The processes included training of SMAGs and procurement of supplies, including bicycles and medicines, and the creation of data collection tools/systems that would facilitate the development of the community Health Management Information Systems (HMIS). These processes were expected to lead to short-term results that were expressed as output indicators, such as numbers of CHWs trained and referrals conducted. It was also assumed that the processes of the intervention would ultimately lead to medium-term outcomes of the intervention based on baseline coverage data, such as the proportion of mothers receiving at least four ANC visits during pregnancy. Finally, the impact was the long-term goal of the project that would include a reduction in neonatal, infant and maternal morbidity and mortality. However, it was also noted that there would be external factors likely to interact with this theory of change.

### Study participants and sampling

Participants engaged in the intervention were purposively sampled for in-depth interviews and focus group discussions. Healthcare providers were purposively selected for in-depth interviews based on their active involvement in maternal and neonatal health as well as in the intervention. Focus group discussions were conducted with women and SMAGs who were purposively selected. The inclusion criteria for focus groups with women were women of reproductive age, with children less than 1 year old and living within the study community during their most recent pregnancy. The SMAGs were included in the study with the help of healthcare workers at the facility level if they were above the age of 18 years, both male and females, working within the communities under study on the implementation of the intervention and living either within or beyond 5 km radius from the health facility. A total of 78 participants were interviewed, 34 SMAGs, 36 mothers and 8 healthcare providers from Samfya and Luwingu districts.

### Data collection and tools

Eight in-depth interviews were conducted with healthcare providers, using qualitative research techniques to explore issues related to the implementation of the intervention, such as referral practices and supervision at the community level. In addition, eight FGDs were conducted, two from each facility. At each of the four facilities, one FGD was with SMAGs and another with mothers. Trained research assistants with experience in qualitative studies collected the data, 1 year after the intervention. Focus group discussions were conducted by a pair of research assistants, who were of the same gender and fluent in the local language (Bemba). One research assistant facilitated the sessions while the other one managed audio recordings and took field notes. The research assistants underwent a 2-day training prior to the data collection and were supervised by one of the co-authors (CJ). The data collection tools were piloted in a similar facility not included in the study (Additional file 1). The average duration of FGDs and KIIs was 45 min. The interviews were delivered on a face-to-face basis, at the health facilities. Informed consent was obtained from all the participants, and digital voice recorders were used to document the interviews and discussions.

### Data management and analysis

Recorded data were transcribed verbatim, and translated from Bemba to English, supplemented with field notes. All transcripts were assigned a unique identifier and imported into NVivo 13 for data management and analysis. Data was coded by two individuals, a trained research assistant and one of the co-authors (CJ). An iterative inductive thematic approach [24] was used through repeated rounds of reading and re-reading to clarify coding differences and to ensure consistency for subsequent analyses. Coders first independently listened to some recordings, reviewed a sample of the transcripts and began to formulate draft codes and themes. The researchers then met after coding the first six interviews to discuss the coding. Discrepancies were discussed until consensus was reached. Coding meetings with the research team and an experienced research assistant were held every week to create a mutual understanding of codes and refine the coding framework. The two coders examined and assigned sections of text to codes, representing themes or subthemes. Extracts of data were coded and memos were written to record emerging impressions of the data. Coded data extracts were further discussed among all the authors and merged into categories before refining them into themes. To further verify our results, we returned to the raw data. To enhance study validity, triangulation of different data sources (FGDs and IDIs) between different respondent groups was done by cross-examining the data [25, 26]. Triangulation is a recognised method to increase the credibility of data analysis [25]. This was achieved through data triangulation whereby the perspectives of the different respondent groups were explored. We also maintained a detailed audit trail of all decisions through a codebook, coding discussions and meetings.

## Results

A total of 78 participants were interviewed, 34 SMAGs, 36 mothers and 8 healthcare providers from two rural districts. The majority of the SMAGs were female (59%) with an age range of 24 to 71 years. All the SMAGs (100%) received training on safe motherhood skills before the intervention and were working in the community. Three out the eight healthcare providers (38%) were female; one of them was a community health assistant and another a classified daily employee. The mothers’ age range was 19 to 46 years; none of them reported that they have never been to school, and the majority (64%) had not completed primary education.

### Thematic areas

**Fig. 3:**
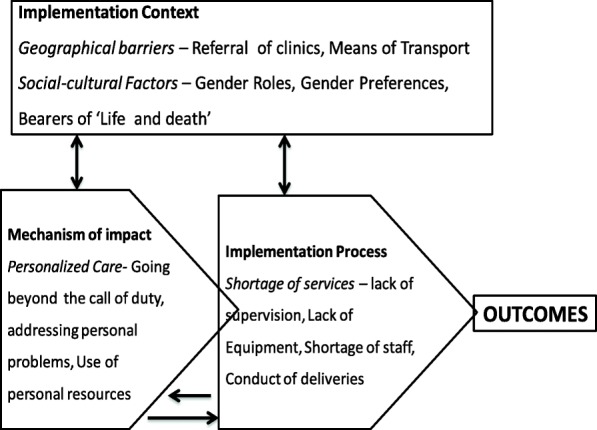
Summary of the findings based on the key constructs of the MRC framework

On the whole, SMAGs were able to implement the intended intervention, by identifying pregnant women and encouraging them to attend ANC visits, assisting with birth preparedness plans and clinic attendance for skilled birth assistance as well as postnatal care. In addition, SMAGs were able to refer women with complications in pregnancy and following delivery, as well as doing follow-up visits for those under their care and submitting the necessary written reports. However, the following themes under the three major constructs of implementation process were found to potentially influence the implementation of the intervention and likely to explain the failure to meet outcome targets: service shortages, geographic barriers, socio-cultural factors and implications of personalising care (see Fig. 3).

### Implementation processes

#### Service shortages

##### Lack of supervision

Most SMAGs expressed concern over lack of supervision and refresher courses from the health facility and district health staff. They indicated that supportive supervision was only provided in the initial phases of the SMAG programme, which was demotivating and a cause of dropout for some SMAGs.

When the programme started in 2013, they used to come and check on us. But nowadays we just work with the staff. Otherwise, there is no support provided to us, and others end up withdrawing. (Mother: FGD3:1)

##### Lack of equipment

SMAGS also reported that lack of protective clothing such as gumboots, raincoats and torches made movements at night or during the rainy season challenging. These additional supplies would have assisted SMAGs to work more efficiently through the nights and rains.

Yes ......these are things that we lack and without these, our work is very challenging, things like raincoats, torches because they [patients] come to wake us up in the night to escort them. (SMAG: FGD2: 2:7)

##### Shortage of staff

The respondents reported that lack of skilled health personnel in some facilities made the work of SMAGs more difficult in that some women in the community did not see any benefit of being referred to a health centre where there was no skilled personnel.

We have a problem because some women do not see any need of going to the facility only to be attended by someone who is not a nurse, and especially a young man. (SMAG: FGD2:2:2)

##### Conduct of deliveries

Discussion with respondents revealed that SMAGs conducted deliveries in the health facilities. According to most healthcare providers, the SMAGs were a relief in facilities when healthcare providers were overwhelmed with work or not available.

Ah for delivery, usually they [SMAGs] refer clients to clinic, but when I am not at the facility since I am alone, they [SMAGs] do conduct deliveries. (HCW: IDI1:1)

SMAGs were seen to have taken over the roles of TBAs, which made it harder for healthcare providers and community members to shift their expectations, and in fact, expected SMAGs to do the work of the TBAs.

It is hard to differentiate the care given by SMAGs from that of TBAs because it is like their roles have been mixed… yes uh, the SMAGs are doing the roles of the TBAs. (HCW: IDI2:1)

### Implementation context

#### Geographic barriers

##### Referral to clinics

Distance to the health facility, poor road conditions and inadequate transport to the health facility emerged as prominent challenges that hindered SMAGs’ ability to refer women to the health facilities for skilled care.

The points that my sisters have said are true, we have a challenge here because some of our areas are very far......... others have to cross two streams, they live very far where there is no SMAG. So the point here is that we have to sacrifice to go and see them, and if you don’t have a bicycle to use it is a problem. (SMAG: FGD2:3:3)

##### Means of transport

The need for SMAGs to transport pregnant women to the health facility, and the difficulty thereof, hampered their ability to promote skilled deliveries in health facilities. A female SMAG respondent confirmed that some communities were more than 4 h walk to the health facility.

Some of them are within the clinic catchment area of Ndoki, but others take about 4 hours walking to reach here so if there is no bicycle you walk with a pregnant woman, imagine the challenge. (SMAG: FDG2: 1:1)

All the SMAG respondents stated that bicycles, a major means of transport that was used in the communities, were either not available or were inadequate since these were shared among all the SMAGs in the community.

#### Socio-cultural factors

##### Gender roles

An intended gender-neutral role for SMAGs, which entails similar roles between male and female SMAGs, was reported as a challenge for effective provision of referral services by the SMAGs. Male SMAGs expressed concern that some mothers, particularly those unmarried, found it difficult to seek care from a male SMAG on pregnancy or childbirth-related issues and needs. Male SMAGs, therefore, preferred to send female SMAGs to young unmarried mothers to identify problems.

Just as someone said, most women who get pregnant and are not married feel shy to come and tell a male SMAG, so we send a female SMAG to see her. Then that female SMAG will now come and tell us what the problem is. (SMAG: FGD1:2:8)

##### Gender preferences

Healthcare providers confirmed the SMAGs’ assertions over gender preferences by mothers and that male referrals are few and far in between, which may indicate the compromised effectiveness of male SMAGs in their role compared to female SMAGs.

Yes, in fact even referrals that I usually receive most of them are those that are referred by female SMAGs, as for male SMAGS, I think it takes a year ah...ah it can happen once a year. (HCW: 1DI2:4)

##### Bearers of ‘life and death’ reports

When SMAGs escorted a woman for facility deliveries, they had to wait for the women to deliver. The long hours and sometimes days of waiting were a challenge to the roles of SMAGs. When asked why they waited, most SMAGs explained that communities viewed them as bearers of ‘life and death’ news.

Concerning waiting for the woman until she is discharged, you get worried because pregnancy is a matter of life and death. So this makes us wait until a woman delivers because once the family members hear that the woman has delivered, they become relieved. That is why she is saying we wait. (SMAG: FGD1:2:6)

### Mechanism of impact

#### Personalising care

##### Going beyond the call of duty

SMAGs provided personalised care beyond their role in the intervention. The personalised care included among others house chores and marital counselling. These extended roles were outside their prescribed scope of work but appreciated by the pregnant women whose physical conditions necessitated support. One of the mothers said:

When they come to visit us they bring us food and even draw for [us] water just like that. (Mother: FGD2:1:3)

##### Addressing personal problems

SMAGs were regarded as individuals who could not only handle some of their health needs but also mothers’ personal problems. Some mothers indicated that the trust they had in SMAGs motivated them to seek advice from the SMAGs. One of the mothers had this to say:

We trust them, which is why we go to them. Even if you are quarrelling at home, we get up and go to the SMAG. When you are pregnant, and tell them listen, my husband has failed to prepare. Come and teach him so that he knows how to prepare the clothes for the baby, because others are drunkards. (Mother: FGD3:9)

##### Use of personal resources

Sometimes, the SMAGs had to use their personal resources to meet some costs such as transport costs to get the mothers to the health facilities. Most women and their families could hardly afford decent clothing for their newborn baby because of poverty.

Sometimes, a woman and her husband may not even have things for the baby, so you the SMAG have to give them a chitenge or a nappy. So that money has to be paid for by us SMAGs just to help our friends to deliver on time. (SMAG: FGD1:1:6)

In such circumstances, the SMAGs would take up the responsibility of securing clothing for the newborn baby, and related requirements, just to overcome the barrier and get women to the health facility. These expenses made it difficult for the SMAGs, as they themselves were also poor.

## Discussion

The current study, a process evaluation approach, was designed to provide insights into explanatory mechanisms for the variations observed between actual and targeted outcomes for MNH services, ANC and SBA, following a SMAG community-based intervention in Zambia. We found, firstly, that SMAGs implemented much of the intervention as was intended, particularly education of women and referral to health facilities for ANC, SBA, PNC and complications. Secondly, SMAGs went above and beyond their prescribed roles to assist women with household chores and personal problems and even used their own resources to support needy households, so as to enhance the success of their work. Thirdly, SMAGs reported what could be regarded as deficiencies in the intervention itself, due to poor ongoing support, inadequate supplies and lack of effective transportation needed to enable success in their work, leading to attrition of SMAGs from the intervention. Fourthly, the beneficiaries did not perceive the intervention to be gender-neutral as was assumed during intervention design; in that, mothers preferred to disclose their health issues to female SMAGs rather than male SMAGs, thereby compromising the intensity of the intervention. Lastly, factors external to the intervention may have compromised the results, particularly the inadequacy of health services in facilities where SMAGs referred mothers, made worse by the long distances and difficult terrain women had to travel in the company of SMAGs.

The health facility-related service deficiencies led SMAGs to engage in the unintended role of conducting deliveries. Some SMAGs were also known to be TBAs, whose birth attendance activities have been terminated despite their many years of experience in assisting births in the communities. Previous studies have indicated that SMAGs were chosen from the existing TBAs and other community health volunteer [27, 28]. However, health workers allowed SMAGs known to have worked as TBAs to conduct deliveries in the health facility when facilities experienced absenteeism, shortages or work pressure. The unintended consequence of hiring previous TBAs meant that SMAGs could conduct deliveries in health facilities, but these were not recognised as skilled attendance, even though some of these deliveries may have been performed under the supervision of nurses. Such implementation mechanisms could explain the failure of the SMAG intervention to meet the set targets for SBA. The role confusion between activities of SMAGs and TBAs should be considered in future interventions to avoid unintended consequences [29]. Enhancing strategies that provide awareness of the specific roles of CHWs is critical to guide expectations of communities and healthcare providers towards the specific services in community-based interventions [30]. We also suggest the critical need of ensuring availability of skilled personnel in health facilities to avoid similar unintended consequences of SMAGs [31].

Further, the findings in this study are consistent with the studies in Zambia and elsewhere [28, 29], which have highlighted the importance of recruiting SMAGs within their communities where they are trusted and preferred. The SMAGs’ unique position and moral authority within these communities had the potential to influence outcomes to the intervention positively [28, 29, 32–34] given their existing social networks [28, 29] and inclination to be natural helpers through personalised care provision [35]. However, provision of personalised care by SMAGs also meant going beyond their scope and using their own personal resources, an indication of the inadequacy of the health system and a threat to optimal provision of referral services, a key component of the SMAGs intervention. Use of personal resources by CHWs has been reported in other rural studies that also involved referral or community members to health facilities [36]. For the SMAG intervention to function and be sustainable, empowering CHWs with financial incentives appears to be critical in order to enable them to effectively provide their services and meet needs of community members in rural and poor communities [37, 38]. The remoteness of rural communities has been reported in other studies as a barrier to mothers’ ability to reach the healthcare facilities [5, 39–41]. These findings suggested the need for effective and sustainable means of transport in community-based interventions designed to improve access by linking communities to the health system through SMAGs [42].

In these remote areas, women found it easier to disclose pregnancy-related issues to female SMAGs than male SMAGs, as healthcare occurs within cultural-bound norms and sensitive socio-cultural factors, often more common in remote rural communities [43, 44]. The findings thus suggest a need for gendered SMAG roles [45] and male-female task sharing functions if MNH services are to be optimised for marginalised women in remote and hard-to-reach areas. Feldhaus et al. argued that pairing male and female SMAGs may potentially address and accommodate gender preferences among SMAGs for pregnancy-related issues [46]. All in all, these findings highlight the interrelationships between the contextual factors and the implementation processes of the intervention.

However, this study has potential limitations that should be noted. Firstly, the collection of data for this process evaluation was conducted after the outcome analysis, which may have been compromised by recall bias in retrospectively reconstructing events, and a prospective process evaluation design would have been more preferable. Secondly, self-reported accounts of intervention fidelity in this study are prone to bias and may have lower reliability than observational measures [47]. However, the adoption of a data triangulation approach was intended to allow for validation of findings from different sets of participants. Thirdly, data for this study was collected from respondents who were still active as SMAGs, and therefore not representative views from inactive SMAGs.

## Conclusion

In conclusion, we found evidence suggesting that although SMAGs continue to play pivotal roles in contribution towards accelerated coverage of maternal and neonatal health services among hard-to-reach populations, they are unable to meet some of the critical sets of MNH service targeted indicators. Complexities of the implementation mechanisms coupled with the presence of differential setting specific social, cultural and geographical contextual factors could partly explain this failure. More specifically, we think that deficiencies in the implementation mechanisms and the factors external to the SMAG intervention such as the health services inadequacy of skilled healthcare providers in facilities where SMAGs referred mothers as well as poor geographical access may have led SMAGs to engage in the unintended role of conducting deliveries. This could have compromised the performance of the SMAGs and led to the failure to meet the set targets for key MNH service indicators among women in the most rural and remote communities. These observations point to limitations in past and existing efforts to improve MNCH health service delivery. There is thus need to innovate and re-package existing implementation strategies, such as recruiting adequate health care providers and ensure adequate provision of logistics and equipment, so as to help SMAGs and any other community health systems champions to effectively respond to MNH needs of most-at-risk women and promote universal health coverage targeting hard-to-reach groups. If this is done correctly, policy and programme managers will be armed with systems and strategies that adequately and appropriately match the complex nature of the implementation mechanisms and external contextual factors that hinder successful implementation of community-based interventions such as SMAGs in hard-to-reach areas.

## Additional file

Additional file 1: Discussion and interview guides. (DOCX 45 kb)

## Abbreviations

ANC: Antenatal care
CHW: Community health worker
FGD: Focus group discussion
HPP: Health for the poorest populations
IDI: In-depth interview
MNH: Maternal and neonatal health
MRC: Medical research council
PNC: Postnatal care
SBA: Skilled birth attendants
SMAGs: Safe motherhood action groups
