# Supplementary material for: Implementation of a community-based intervention in the most rural and remote districts of Zambia: a process evaluation of safe motherhood action groups
Source: Implement Sci. 2018 May 31;13:74. doi: 10.1186/s13012-018-0766-1 (PMC5984469; doi:10.1186/s13012-018-0766-1)
Supplement: Supplementary file 1 — Discussion and interview guides. (DOCX 45 kb) [file 13012_2018_766_MOESM1_ESM.docx]

# DISCUSSION AND INTERVIEW GUIDES

| **Perceptions, experiences and preferences in community-based interventions and the role of the Safe Motherhood Action Groups in the remote and marginalised communities of Zambia** | | |
| --- | --- | --- |
| **Discussion Guide with Mothers** | | |
| **TOPIC** | **QUESTION AND ACTIVITY GUIDE** | **PROBES** |
| Background | 1.1 Could you please tell me about yourselves | Age, location, marital status, number of children, level of education |
| Women’s’ Awareness of existence of SMAGs and accessibility of their services | - 1. Have you ever heard of community volunteers that provide maternal and neonatal health services in this community?   2. Which Community Health Volunteers do you have in this community?   *Am now going to tell you about the care given by the SMAGs. The SMAGs are a group of community members that provide health education to mothers in the community on safe pregnancy and delivery particularly on danger signs and care during pregnancy; planning for safe pregnancy and delivery. In addition, SMAGs guide mothers on the identification of systems within the community that will increase access to care. Furthermore, SMAGs provide immediate new-born, care of the new-born in the first 30 days and postnatal care for mothers and new-born.*  2.2 Do you have such a group in this community?  2.3 How did you come to know about the existence of SMAGs and their services  2.4 How long have you had SMAGs in this community  2.5 Were you ever attended by a SMAG during your last pregnancy | Do you have any of these; SMAGs, TBAs or CHWs in this community  Probe: Tell me, Which ones have you heard about |
| Women’s Perceptions of the role of SMAGs | 3.1 What role do SMAGs play during ANC  3.2 What role do SMAGs play during delivery?  3.3 Can you describe the work that the SMAGs do during Postnatal care?   - 1. Do you think SMAGs are available when you need their services   3.5 How does the care given by the SMAGs compare with the care given by other community health workers or Traditional Birth Attendants?   - 1. From which community Health Volunteers do most pregnant women seek care and information from? | Probe. In the community  Probe. At the facility |
| Women’s experiences with services offered by SMAGs | 4.1 What are your impressions about the role of SMAGs in relation to ANC, Deliveries, PNC  4.2 Do you remember anything about the service you receive from a SMAG  4.3 Can you tell me what happened when you had a SMAG at your service  4.4 What did you like about the service provided by a SMAG  4.5 Could you tell me how you were treated by a SMAG?  4.6 Were you you satisfied with the care you received, if yes why, if no why | Probe: During ANC  Probe: During Deliveries  Probe: During PNC |
| Barriers and facilitators to accessing services from SMAGs | 5.1 What are the concerns that you and your peers have with regards to the care provided by the SMAGs? *(Take time to list down all the concerns and complaints)*  5.2 What are the challenges you face with access and services from SMAGs  5.3 What makes it easy for you to access the services from the SMAGs  5.4 What is good about the services provided by SMAGs  5.5 What motivates mothers seek antenatal care from the most preferred CHW. Give reasons  5.6 What benefits have you experienced from the care provided by the SMAGs? | Gender, Distance, costs, attitude  Probe: in your homes  in the community?  At facility |
| Women’s preferences with services provided by SMAGs | 6.1 Would you prefer accessing the services from SMAGs than from TBAs, or CHWs  6.2 What would you prefer or what service would you rather receive from a SMAG  6.3 What care would you prefer the SMAGs to provide to the mothers in the community. *(Take time to list down all the concerns and complaints)*  Probe: Do you think the care provided by the SMAGs is adequate enough in improving access to skilled health care? If so, Why? If not, why not?  6.4 What other services do you think should be provided SMAGs? |  |
|  | 7.1 Do SMAGs refer women to the health facility  7.2 Tell me, how do they refer women to the health facility  7.3 Are there any challenges in the way they refer women? |  |
| Conclusion | 8.1 What strategies can you recommend to government/Health facility over the care provided by the SMAGs to overcome the barriers that mothers face in utilising skilled deliveries and postnatal care?  8.2 Before we close up our discussion, what do you think are the most important issues to be addressed about the care provided by the SMAGs?  8.3 Is there anything else you would like to tell me about the care provided by the SMAGs? |  |

| **Perceptions, experiences and preferences in community-based interventions and the role of the Safe Motherhood Action Groups in the remote and marginalised communities of Zambia** | | |
| --- | --- | --- |
| **Discussion Guide (Community Health Volunteers – SMAGs)** | | |
| **TOPIC** | **QUESTION AND ACTIVITY GUIDE** | **PROBES** |
| Background | 1.1 Could you please tell me about yourself and your family | Age, location, marital status, level of education |
| Women’s’ Awareness of existence of SMAGs and accessibility of their services | 2.1 Where do women in this community seek maternal and neonatal health care?  2.2 Who are the main providers of Antenatal care within the community?  2.3 From which community Health Volunteers do most pregnant women seek care and information from?  2.4 What motivates mothers maternal health services preferred CHV  health care  P: Give reasons  **2.6** Which Community Health Volunteers do you have in this community?  Probe: Do you have any of these. SMAGs, TBAs or CHWs in this community  Probe: What do each of these volunteers do in this community? | Probe: ANC, Deliveries, PNC |
| Perceptions on the role of SMAGs | 3.1 What role do SMAGs play in maternal and neonatal health services  3.2 What motivates mothers seek antenatal care from the most preferred CHW  Give reasons   - 1. Do you think SMAGs are available when you need their services   2. How does the care given by the SMAGs compare with the care given by other community health workers or Traditional Birth Attendants? | Probe: During ANC, Deliveries, PNC  Probe: In the at individual, community, at the health facility level |
| Women’s experiences with services offered by SMAGs | 4.1 what services have you provided in the past to the following;  Women,  Men  Adolescents  4.2 What are your impressions about the services you offer to the above stated categories?  4.4 Are women comfortable with SMAGs providing the services to you? If yes, why? If not, why?  4.5 What benefits do women get from the care provided by the SMAGs?  4.6 What are the concerns that women have with regards to the care provided by the SMAGs? ***(Take time to list down all the concerns and complaints)*** | Will you continue to use the services or not  Communication, information, fair treatment |
| Barriers and facilitators to accessing services from SMAGs | 5.1 What are the concerns that you have with regards to delivery of services *(Take time to list down all the concerns and complaints)*  5.2 What are the challenges you face with the delivery of services  5.3 What makes it easy for you deliver the services to women  5.4 What is good about the services provided you provide  5.5 What benefits have you experienced from the service you provide as SMAGs |  |
| Refereal System | 6.1 Do you refer women to the health facility  6.2 Tell me, how do you refer women to the health facility and when do you do it  6.3 Are there any challenges you face in referring women to the facility |  |
| Women’s preferences with services provided by SMAGs | 7.1 What would you prefer or what service would you rather want to provide  7.2 What care would you prefer the SMAGs to provide to the mothers in the community. *(Take time to list down all the concerns and complaints)*  7.3 Do you think the care provided by the SMAGs is adequate enough in improving access to skilled health care? If so, Why? If not, why not?  7.4 What other services do you think should be provided SMAGs? |  |
|  | 8.1 What strategies can you recommend to government/Health facility over the care provided by the SMAGs to overcome the barriers that mothers face in utilising skilled deliveries and postnatal care?  8.2 Before we close up our discussion, what do you think are the most important issues to be addressed about the care provided by the SMAGs?  8.3 Is there anything else you would like to tell me about the care provided by the SMAGs? |  |

| **Perceptions, experiences and preferences in community-based interventions and the role of the Safe Motherhood Action Groups in the remote and marginalised communities of Zambia** | | |
| --- | --- | --- |
| **Interview Guide with Health Care Providers** | | |
| **TOPIC** | **QUESTION AND ACTIVITY GUIDE** | **PROBES** |
| Background | 1.1 Could you please tell me about yourself and your family | Age, location, marital status, level of education |
| Women’s’ Awareness of existence of SMAGs and accessibility of their services | - 1. Have you ever heard of community volunteer that provide maternal and neonatal health services in this community?   2. Which Community Health Volunteers do you have in this community?   *Am now going to tell you about the care given by the SMAGs. The SMAGs are a group of community members that provide health education to mothers in the community on safe pregnancy and delivery particularly on danger signs and care during pregnancy; planning for safe pregnancy and delivery. In addition, SMAGs guide mothers on the identification of systems within the community that will increase access to care. Furthermore, SMAGs provide immediate new-born, care of the new-born in the first 30 days and postnatal care for mothers and new-born.*  2.3 Do you have such a group in this community?  2.4 How did you come to know about the existence of SMAGs and their services  2.5 How long have you had SMAGs in this community  2.6 Were you ever attended by a SMAG during your last pregnancy | Do you have any of these; SMAGs, TBAs or CHWs in this community  Probe: Tell me, Which ones have you heard about |
| Women’s Perceptions of the role of SMAGs | 3.1 What role do SMAGs play during ANC  3.2 What role do SMAGs play during delivery?  3.4 Can you describe the work that the SMAGs do during Postnatal care?   - 1. Do you think SMAGs are available when you need their services   3.6 How does the care given by the SMAGs compare with the care given by other community health workers or Traditional Birth Attendants?   - 1. From which community Health Volunteers do most pregnant women seek care and information from?   Probe: What do the SMAGs do in this community?  3.8 What motivates mothers seek antenatal care from the most preferred CHW  Give reasons | Probe. In the community  Probe. At the facility  Probe. In the community  Probe. At the facility |
| Women’s experiences with services offered by SMAGs | 4.1 What are your impressions about the role of SMAGs in relation to ANC, Deliveries, PNC  4.2 Do you remember about the service you receive from a SMAG  4.3 Can you tell me what happened when you had a SMAG at your service  4.4 What did you like about the service provided by a SMAG  4.5 Could you tell me how you were treated by a SMAG? Where you satisfied with the care you received | Will you continue to use the services or not |
| Barriers and facilitators to accessing services from SMAGs | 5.1 What are the concerns that you and your peers have with regards to the care provided by the SMAGs? *(Take time to list down all the concerns and complaints)*  5.2 What are the challenges you face with access and services from SMAGs  5.3 What makes it easy for you to access the services from the SMAGs  5.4 What is good about the services provided  5.5 What benefits have you experienced from the care provided by the SMAGs? | Gender, Distance, costs, attitude, Presence of staff. |
| Refereal System | 6.1 Tell me, how do SMAGs refer women to the facility  6.2 Do you refer women to the SMAGs  6.3 If you do so, how do you do it, and when do you do it |  |
| Women’s preferences with services provided by SMAGs | 7.1 Would you prefer accessing the services from SMAGs than from TBAs, or CHWs  7.2 What would you prefer or what service would you rather receive from a SMAG  7.3 What care would you prefer the SMAGs to provide to the mothers in the community? *(Take time to list down all the concerns and complaints)*  7.4 Do you think the care provided by the SMAGs is adequate enough in improving access to skilled health care? If so, Why? If not, why not?  7.5 What other services do you think should be provided SMAGs? |  |
|  | 8.1 What strategies can you recommend to government/Health facility over the care provided by the SMAGs to overcome the barriers that mothers face in utilising skilled deliveries and postnatal care?  8.2 Before we close up our discussion, what do you think are the most important issues to be addressed about the care provided by the SMAGs?  8.3 Is there anything else you would like to tell me about the care provided by the SMAGs? |  |
